# Multi-Criteria Evaluation and Sensitivity Analysis for the Optimal Location of Constructed Wetlands (METland) at Oceanic and Mediterranean Areas

**DOI:** 10.3390/ijerph18105415

**Published:** 2021-05-19

**Authors:** Lorena Peñacoba-Antona, Montserrat Gómez-Delgado, Abraham Esteve-Núñez

**Affiliations:** 1 IMDEA Water Institute, Av. Punto Com, 2, Parque Científico Tecnológico, 28805 Alcalá de Henares, Madrid, Spain; lorena.penacoba@imdea.org; 2 METfilter S.L., Autovía A49 Sevilla-Huelva Km 28, 41820 Carrión de los Céspedes, Sevilla, Spain; 3 Department of Analytical Chemistry, Physical Chemistry and Chemical Engineering, University of Alcalá, Ctra. Madrid-Barcelona Km 33.600, 28871 Alcalá de Henares, Madrid, Spain; 4 Department of Geology, Geography and Environment Science, University of Alcalá, Ctra. Madrid-Barcelona Km 33.600, 28871 Alcalá de Henares, Madrid, Spain; montserrat.gomez@uah.es

**Keywords:** wastewater treatment, nature-based solution, constructed wetland, METland, Geographical Information System, Multi-Criteria Evaluation, Global Sensitivity Analysis

## Abstract

METland is a new variety of Constructed Wetland (CW) for treating wastewater where gravel is replaced by a biocompatible electroconductive material to stimulate the metabolism of electroactive bacteria. The system requires a remarkably low land footprint (0.4 m^2^/pe) compared to conventional CW, due to the high pollutant removal rate exhibited by such microorganisms. In order to predict the optimal locations for METland, a methodology based on Multi-Criteria Evaluation (MCE) techniques applied to Geographical Information Systems (GIS) has been proposed. Seven criteria were evaluated and weighted in the context of Analytical Hierarchy Process (AHP). Finally, a Global Sensitivity Analysis (GSA) was performed using the Sobol method for resource optimization. The model was tested in two locations, oceanic and Mediterranean, to prove its feasibility in different geographical, demographic and climate conditions. The GSA revealed as conclusion the most influential factors in the model: (i) land use, (ii) distance to population centers, and (iii) distance to river beds. Interestingly, the model could predict best suitable locations by reducing the number of analyzed factors to just such three key factors (responsible for 78% of the output variance). The proposed methodology will help decision-making stakeholders in implementing nature-based solutions, including constructed wetlands, for treating wastewater in rural areas.

## 1. Introduction

The importance of water resources has promoted the development of innovative technologies to reduce water consumption. Different strategies were implemented for sustainable water resources management and integral treatment, in order to improve the quality and availability conditions [1]. The governments have implemented policy measures for enhancing water availability with reduced pressure on existing freshwater resources. One of the main lines of action is to optimize wastewater treatments (WWTs), applying new technologies based on economic and environmental sustainability principles. Standard WWTs are not viable in small settlements, isolated dwellings and work centers, due to their decentralized location, the limitation of economic resources and the lack of availability of specialized personnel in many cases. Therefore, it is essential to promote a low-cost system to treat the wastewater (WW) of decentralized population centers, where conventional WWTs are unbearable financially. As a result, most of the current WWT systems were developed and divided into two treatment groups: energy-based solutions commonly cataloged as intensive (extended aeration activated sludge, membrane bioreactors) and nature-based solutions denominated as extensive (vegetation filters, stabilization ponds or CW). Specifically, CW is defined as a WWT system, consisting of shallow, plant-based lagoons, where purification is a set of biological, physical and chemical processes that seek to mimic the conditions presented in the natural wetlands [2,3,4]. Thus, CW are based on three elements: plants (oxygen supply and nutrient absorption), microorganisms (degradation of organic matter and other pollutants) and substrate (hydraulic conductor and filter medium) [5,6]. 

### 1.1. Bioelectrochemical-Assisted Constructed Wetlands (METland) 

**Figure 1:**
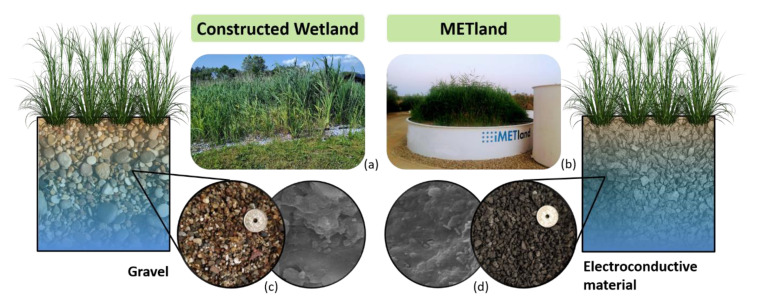
Images of real scale standard CW (**a**) and METland (**b**) including a Scanning Electron Microscopy viewof the biofilter material: gravel (**c**) and electroconductive material (**d**).

Standard design for constructed wetland has been recently altered by the integration of biocompatible electroconductive materials typically used in Microbial Electrochemical Technologies (METs). The new born technology (Figure 1) can be considered a bioelectrochemical-assisted constructed wetlands also named in recent literature as METlands [7,8].

The main feature of METlands is the electroconductive material used as a biofiltering substrate, key for promoting the growth of electroactive bacteria such as those of the genus *Geobacter* [9]. Indeed, *Geobacter* strains are capable of exchanging electrons with electroconductive materials [10] in order to generate electrical current or to perform direct electron interchange with other bacteria [11]. Originally, METland were designed to operate under flooded conditions and short-circuit mode as “snorkel” electrodes [9]. Thus, the natural redox gradient between the anoxic bottom and the naturally oxygenated surface greatly enhanced microbial oxidative metabolism for removing organic pollutants [9] including pharmaceuticals micropollutants [12]. The electron flow along the METland bed was demonstrated by measuring the profile of electric potential along long distances [13,14]. Although most of the MET-based applications are classically operated under anoxic conditions to avoid oxygen competition with anodic reactions, METland has been recently proved to be effective even under down-flow aerated mode [15,16] where nitrification reactions are favored. Full scale METland units have been already constructed at 0.4 m^2^ per person equivalent and demonstrated to be sustainable according to a recent Life Cycle Analysis [16] using different electroconductive granular material like electroconductive coke [17] or more sustainable materials like electroconductive biochar (ec-biochar) obtained after wood pyrolysis at high temperature [18,19]. Such designs were successfully validated at different geographical locations (Spain, Denmark, Argentina), in the context of the H2020 iMETland project (www.imetland.eu accessed on 5 April 2021). The largest METland system constructed so far (Natural Park of Cabo de Gata, Spain) is able to treat WW from 1000 pe in a camping site (Esteve-Núñez personal communication). 

As with any nature-based solution, METland performance is related to the habitat where it is applied. Thus, its proper implementation would surely benefit from having predictive methodologies for finding optimal locations. 

### 1.2. Multi-Criteria Evaluation for Finding the Optimal Location

Finding an optimal location for implementing a nature based-solution requires a methodology based on the use of GIS and MCE techniques. The analysis considered environmental and socio-economic variables, to make the study as complete as possible for global replication. Variables included in the study of a nature-based solution like METland should be evaluated by the experts, to determinate the factors that influence the location.

MCE is a set of techniques that allows the analysis of several alternatives in order to achieve one or more objectives [20]. This procedure facilitated decision-making based on a set of criteria and constraints [21]. MCE technical began to develop in the 1970s, mainly in the area of economics [20], developing to this day in a multidisciplinary way as a spatially explicit decision-support tool in combination with GIS [22,23,24] for identifying suitable areas that satisfy several criteria simultaneously. The phases of the procedure are: define the problem, determine objectives and alternatives, select the criteria (factors and constraints) in the light of which alternatives will be evaluated, determine the weights to be assigned to the previously standardized factors, application of the selected MCE method and results analysis [25,26]. Among the wide variety of MCE techniques, one of the more common methodology in this field is Analytical Hierarchy Process, AHP [27], due to its capacity to desegregate the decision problem in some criteria groups, simplifying the final evaluation [28]; and also because it is easy-to-understand and intuitively appealing to decision-makers [23]. 

The combination of MCE and GIS leads to solving a diversity of complex geospatial problems [29,30], discriminating against the most suitable alternatives to develop a particular activity (biomass plants, landfills, agriculture irrigated land, green infrastructures, delimitation of protected areas, wind farm projects, among many others) and being able to simulate different scenarios [23,31,32,33,34,35,36,37].

MCE and GIS have been previously implemented in the water sector, mainly focus on rainwater collection [38], sewage treatment plants [39], decentralized WWTs [40], nature-based solutions for treating WW [41,42,43,44,45] and wetland creation or restoration [46,47,48,49]. To the best of our knowledge, no studies related to the CW for treating WW are available based on the methodology proposed. The available literature shows that some methods are more suitable than others depending on the problem faced, the typology of the input data, the scale and other features. 

### 1.3. Sensibility Analysis Associated with Spatially Explicit MCE Techniques

The models described were normative models, based on expert experience. Therefore, the need to carry out model validation processes has generated a multidisciplinary interest in the application of Sensibility Analysis (SA). These methods allow to determine quantitatively the influence of each of the parameters in the variation of the results, individually and in association with others. Regarding MCE techniques, the objective of the SA is to determine how the final model is influenced by variations in weights and input factors [34]. The dependence between alternatives, weights and the model defines the decision [50]. The final solution could be very susceptible to any small changes in the input data, or, on the contrary, could be very robust so that it is not affected by variations [51]. It should be noted that one of the targets of SA applied in MCE techniques is to simplify models, allowing for optimization of resources, such as time, money and effort that comes with the acquisition of data and creation of model factors [52]. 

Small communities are seeking decentralized and sustainable wastewater treatments, and constructed wetlands like METland are appropriate solutions due to their versatility and low operation cost. The novelty of our work is to implement for first time a prediction methodology through MCE-GIS tools to determine the optimal location of such technologies in oceanic and Mediterranean locations. A sensitivity analysis was conducted for the determination of the most influential factors. Finally, a model optimization was accomplished using the three most influential factors, providing a breakthrough for planning wastewater treatments. Therefore, our scientific contribution facilitates decision-making tools to implement nature-based solutions and reduce the resources used by stakeholders.

## 2. Materials and Methods 

### 2.1. The Study Area

**Figure 2:**
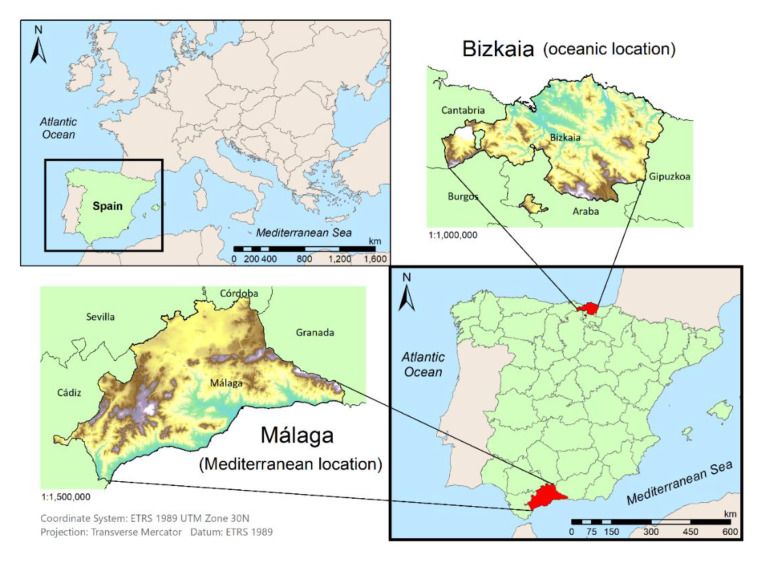
The study area, Bizkaia (oceanic location) and Málaga (Mediterranean location).

Two different study areas (Figure 2) were selected for applying the methodological proposal with the aim to develop a valid procedure to be extrapolated into other locations. According to this premise, two Spanish provinces with different characteristics were chosen. Firstly, Bizkaia (oceanic location) with an area of 2217 km^2^ and a population of 1,152,651 [53]. Secondly, Málaga (Mediterranean location) was selected with an area of 7306 km^2^ and a population of 1,661,785 [53]. The percentage of the population in the capital of each province was similar, 30% in Bilbao (346,843) and 34.58% in Málaga (574,654) [53]. The distribution of the inhabitant within the rest of the province was different among them. In Málaga, the population is mainly concentrated in the metropolitan area of the capital and along the coastal strip, with predominant tourist activity. In Bizkaia, the population is concentrated along the estuary and in isolated villages or houses, with important industrial activity.

Regarding the characteristic climate of the provinces, Bizkaia is defined by an oceanic climate, identified by constant rainfall throughout the year, with temperatures softened by the sea effect ranging between 8 and 10 °C in winter and 18 and 22 °C in summer [54]. Instead, Málaga features a Mediterranean climate, characterized by dry and warm summers with temperatures above 22 °C, and low rainfall concentrated in the colder months, with torrential episodes causing flooding [54]. Based on these data, the climatic and demographic distribution differentiation between the two provinces was corroborated.

### 2.2. Methodology

**Figure 3:**
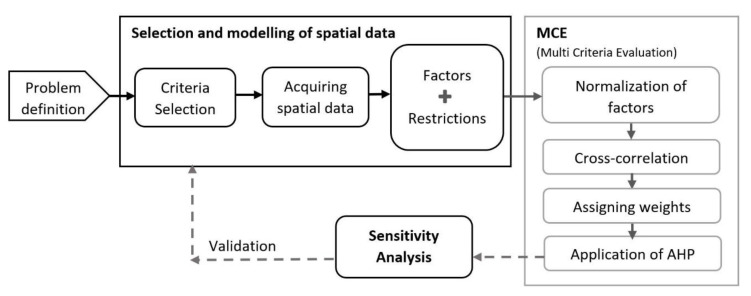
Schematic diagram of the methodological process. The integrated GIS and MCE framework developed in this study using AHP techniques.

The complex geospatial problem to determine the most suitable areas for building METland led to the necessity of using GIS combined with MCE. This union had the advantage of incorporating the knowledge of the decision-makers in the modelling process. In the present study, the location of suitable land for METland and the optimization of the resources involved three main steps (Figure 3). Once the problem was formulated, the first step was to select and prepare the spatial data collected as factors and restrictions. The second step was the implementation of the MCE itself. The third step corresponded to the SA that validates the model and increased the robustness of the results. It must be remarked that in the last step, if the results of the analysis were unsatisfactory, the factors included in the model should be reconsidered. This procedure was followed for both provinces individually, subsequently proceeding to the comparison of the results. 

#### 2.2.1. Multi-Criteria Evaluation Procedure

The constructive cost for METland was determined by the location, volume and concentration of pollutants in the water. The quality and quantity of WW were both difficult to define for large scales, due to the variation between the urban WW in each population center. Thus, the geographical variables for selecting suitable lands for METlands were: land use, climate, orography, demography and the distance to rivers and villages. Specifically, the distance to the discharge point, the population center that produces the WW, and the slope of the ground, mainly influenced the construction cost of the system. Therefore, the modification of these variables affects the budget; construction (length of pipes, excavation, transport of materials and so forth) and operation (pumps, increased in the flowrate, oversized and so forth). 

This study was focused on the location of suitable zones to implement METland on a large scale as a preliminary study. The footprint for the construction of the systems could vary, going from one square meter for one single household up to several hectares for a large city. Therefore, the criteria followed in this study was to select parcels of 25 × 25 m (the raster grid definition) to cover enough area for the average system, so every pixel was then considered as a discrete candidate location. Afterwards, a detailed study should be conducted for setting the correct surface depending on the volume and pollutants of the WW treated. It must be highlighted that the METland system needs lower surface requirements (0.4 m^2^/pe) than standard CW (3–5 m^2^/pe) for treating urban wastewater; therefore, the system easily adapt to the situation with less available surface. 

Geographical data was managed in the reference system ETRS89, projection UTM, zone 30 N. All information was processed using QGIS 2.18.21 [55] for vector information and TerrSet 18.21 from the IDRISI program [56] for all raster processes. The ArcGIS10.4.1 [57] program was used for the design of graphical outputs and maps. 

##### Environmental Factors

Climate information (pluviometry and temperature) was grouped in a database with the geographical location of each weather station of the study areas. Once all stations were georeferenced, several interpolation methodologies were tested with a pixel size according to the Digital Elevation Model (DEM) of 25 m. The interpolation methods carried out were Inverse Distance Weighting (IDW) [58], Triangulated Irregular Network (TIN) [59], Kriging [60] and trend by a global polynomial function [61]. The results of these interpolations were compared with the official map made by the Environmental Information Network of Andalucía [62]. IDW was agreed upon as the best way to interpolate the climatic variables, due to its representativeness with smooth changes and data through the entire territory. Once all the climatic factors were represented, a consultation was made with experts in order to discern which one of the climate variables should be taken into account for the model factors.

1. Temperature. Concerning the temperature, the average was representative of the condition at which the METland will perform over time, especially influencing the growth of vegetation and the operational capabilities [44,63]. For macrophytes (the most common wetland plants) the optimum development temperature was 20 °C and the growth range from 16 to 27 °C. Temperatures above 30 °C and below 10 °C produce vegetative detention [64].
2. Precipitation. The maximum precipitation notably influences the system for two reasons: the increase in the inlet water flow due to runoff and the influence of rain on the plants. Therefore, the existence of torrential rains produces a negative effect associated with a higher volume of water to be treated [65], decreasing the hydraulic retention time and forcing it to enlarge the system area [1].
3. Solar orientation. The sunlight affects the development of vegetation, or more precisely the photosynthesis process. The most suitable orientation in the study area for vegetation was when the slope faces south due to its warmth and luminosity [66].

##### Socioeconomic Factors

On the other hand, the economic resources for treating the WW in most of the small population centers are limited. Indeed, the selected factors are high on capital savings and optimization of the operation of the system.

1. Land use. The adequacy for particular land use to build a CW design like METland was taken into account; for example, forests or crops were less suitable than open spaces with little or no vegetation. Furthermore, the economic cost, environmental impact and social appreciation were considered in the classification. The reclassification of land uses to a quantitative suitability scale of 0 to 10 was performed for each category with a value from 1 (no appropriate) to 10 (very appropriate), summarized in Table 1.
2. Distance to river beds. The distance to the river is a factor that would influence the cost of construction, taking into account that the effluent water of the system would discharge into a river, fulfilling the limits of the current quality regulations [67]. In certain cases, the effluent water could be infiltrated on the ground or evaporated with specific systems such as the willow system.
3. Distance to population centers. The distribution of the population in the study areas was analyzed in order to define the distance to the verified inhabited areas. This variable could be decisive in the location of the CW for several reasons. Firstly, the number of people determines the volume of WW produced. Secondly, the distance from the houses to the CW imposes the length of the conduction which transports the WW. Thirdly, the location of CW close to the population centers could help to change the idea of the sewage treatment plant to an environmentally sustainable garden. For the population layer, census data and cartography were used. The distribution of the census information to spatial units was performed within the dasymetric techniques [68,69,70,71]. Specifically, the Areal Weighting was used, proportionally transferring the information to the area. In this study, the method Filtered Areal Weighting was implemented, in which auxiliary information such as land use or coverage was needed to exclude uninhabited areas from the analysis [72]. It should be mentioned that in this case only the real residential area was considered. Firstly, the spatial location of the buildings of each province was intersected with the Spanish Land Cover Information System (SIOSE, Sistema de Información sobre Ocupación del Suelo en España) [73]. Therefore, all the areas with real homes or constructed areas dedicated to residential use were obtained. Secondly, the census sections with the number of people for each section were downloaded from the last available census (2011) [53]. Based on this data, spatial analysis was performed to determine the number of inhabitants per building based on the population density in each area. Thus, following this procedure, the area of each residential building with the number of inhabitants was obtained for each province. Once demography maps were finalized, it could be observed that the results match what was described in Section 2.1, concluding with a different distribution in each province.
4. Slopes. CW should be constructed on low slope surfaces (from 0 to 15%) to get a gradual flow of WW from the inlet to the outlet and avoid overland flow during rainy seasons. In addition, the cost of earthworks and transport of soil is directly related to the slope [40].

Once the factors that set the guidelines for determining the location of the METland were analyzed, their standardization was carried out in order to be able to apply MCE techniques. For any other nature-based solution, an exhaustive list of influential factors should be addressed. Some of the factors proposed for METland could be adapted to specific conditions (temperature or precipitation) from other nature-based solutions. Afterward, standardization of all factors was performed using fuzzy functions and the output data was byte-formatted, i.e., they will range from 0 (not appropriate) to 255 (very appropriate). Table 2 lists reclassifications, descriptions and databases of the factors.

Another part of the criteria to be considered were those restrictions responsible for specifying whether there was any place in the territory that should be excluded from the analysis. In this case, the areas occupied by rivers, built areas, natural wetlands and water surfaces were considered restrictions, so they were eliminated from the study. 

Once it was confirmed that the criteria were not cross-correlated, a weight for each criterion was obtained via AHP [27]. Furthermore, the relative importance that each criterion had for the decision-making on the final model (Table 3) was also considered. The process followed was based on the phases described below: (1) decision criteria associated with the goal were identified; (2) the factors were placed by levels, from the most general to the most specific, in the case of concern two levels were established, grouping the criteria into environmental and socioeconomic categories; (3) each hierarchical group was weighted from the Saaty peer-comparison matrix [25]; (4) the weights of the levels obtained in each hierarchy were added, thus achieving the global and final weights for each factor of the analysis. Finally, the alternatives were obtained based on the total score achieved; the higher the value, the greater the adequacy [75]. A Weighted Linear Combination MCE was employed to produce the suitability maps. 

#### 2.2.2. Global Sensitivity Analysis

For the model validation, it was proposed to perform a SA in both study areas, in order to delve into the components of the model and the degree of influence in the variation of the results. In the context of SA associated with MCE techniques the approach could be local or global [76,77,78]. The local SA consists in altering one factor each time and leaving the rest fixed [79]. On the other hand, GSA studies the effect of variations on input factors, taking into account the interaction between the different input factors. 

The interaction between factors or weights was not analyzed in all the SA [31,34,80]. For example, the One-At-a-Time approach (OAT) was applied in some studies with a weight variation only for the main criteria [31], obtaining a partial result that may mask the real variability of the final model. Only the GSA obtained the relations between all the parameters [52]. This interaction could be responsible for a percentage of the final model variation and must not be neglected [32]. Therefore, to assess the robustness of MCE results it is important to test the global sensibility, because the interaction between criteria or weights could be sources of uncertainty causing impacts in the model results [79,81]. There are multiple methodologies for the application of a GSA, specifically in MCE-GIS techniques two of them are mainly used, Sobol and Fourier Amplitude Sensitivity Test (FAST), with their extension in E-FAST for GSA [81]. 

The Sobol method was proposed for the GSA, as it is one of the methods based on variance and widely applied in the field of numerical modelling [79]. Another reason for the election of this method was its application to spatial models [50,52,82]. This technique is included in the group of techniques based on variance estimation, decomposing the variability of the outcome and obtaining some measure of sensitivity, not only for every model input (factors and weights), but also for the combinations of them [81]. It is very appropriate for complex geographical models since they are rarely additive and linear, thus it is not sufficient to explore the inputs individually, but also in combination with an increasing level of dimensionality. 

Variance-based SA breaks down the total unconditional variance (V = V(Y) below) of model output Y caused by the changes in z model inputs and apportions into individual factor i (V_i_) as well as i’s combinations with other factors i.e., j, …, z (V_ij…z_) with an increasing level of dimensionality:

(1) $$V=V_{i}+V_{j}+\ldots +{\;V}_{z}+V_{\mathrm{ij}}+\ldots +V_{\mathrm{iz}}+V_{\mathrm{ijz}}+\ldots +V_{\mathrm{ij}\ldots z}\;$$

This variance is further applied to compute with two sensitivity indices for every factor i. The first-order sensitivity index (S_i_) is a measure that quantifies the fractional contribution of the resultant variance of a given factor i taken independently from other factors [81]:

(2) $$S_{i}=\frac{V_{i}}{V}=\frac{V\left[E\left({Y|X}_{i}\right)\right]}{V\left(Y\right)}$$

where Y is the model output, and $V\left[E\left(Y|X_{i}\right)\right]$ is a variance of the expectation of Y conditional on the factor i having a fixed value. If $\left[E\left({Y|X}_{i}\right)\right]$ substantially varies across X_i_, i is regarded as an important factor. S_i_ represents the major contribution of i to V. However, it does not capture the interaction (second- and higher-order) effects between i and the other factors. It can be addressed with the total effect sensitivity index (ST_i_), which quantifies the fractional contribution to V(Y) of a given factor, including all its interactions with other factors [81,83]:

(3) $${\mathrm{ST}}_{i}=1-\frac{V\left[E\left({Y|X}_{\sim i}\right)\right]}{V\left(Y\right)}={\;S}_{i}+S_{\mathrm{ij}}+S_{\mathrm{im}}+S_{\mathrm{ijm}}+\ldots +S_{\mathrm{ijm}\ldots z}$$

Consequently, ST_i_ includes, in one single measure, first-order and higher-order terms that involve i. 

The GSA was performed by SimLab^®^ v.2.2 software [84]. For obtaining first-order sensitivity index (Si) and the total effect sensitivity index (ST_i_) a Monte Carlo simulation sample was used (f independent input factors and X samples) [81]. Thus, the model was run a significant number of times to extract samples from the probability distribution function of each variable. Table 4 details the distribution functions selected for each model input criteria (reflecting the original distribution of the variable as accurately as possible, based on the mean and the standard deviation). The uniform frequency distribution used to represent the weights ranged between ±20% of the original value. 

For each component, the sensitivity index represented the impact of a single factor and the total impact of the interaction of each factor with the rest [85]. Once the samples were generated and the model was executed, the first order and total sensitivity rates were obtained for each component (weights and factors). At the present methodology the interaction between criteria and weight were included in the GSA, obtaining a clear view of the influence of each of them in the final result. This procedure presents clear advantages for achieving more transparency and robustness of the results.

#### 2.2.3. Optimization of Resources

Both the data availability and computational cost should be considered for applying this methodology. The data collection is time-consuming and sometimes is the “bottle-neck” for the MCE process. Therefore, a simple model with fewer parameters and clear representation will be easier to reproduce in other study areas. This statement follows the popular KISS principle (i.e., “Keep It Simple, Stupid”) among modelers [86] which encourages keeping the construction of models as simple as possible. Following this idea the GSA was performed in the studied methodology to set the path to simplify the model. The GSA based in Sobol determines with high precision the factors, weights or interactions that cause a greater influence in the final results. In other studies, the simplification was not possible because of the limited information obtained by the SA [31,33]. For example, Vavatsikos et al. (2020) [45] only obtained the weight variation in the final results, processing different scenarios, but without detecting the percentage variation in the results due to each factor. 

## 3. Results and Discussion

Early adopters of nature-based WW technologies seek methods to find suitable location and minimize the risk of implementing. The goal of our work is precisely to validate a prediction technology in the context of a decentralized WWT, specifically a variety of constructed wetland—so-called METland.

### 3.1. Multi-Criteria Evaluation of Two Independent Areas

**Figure 4:**
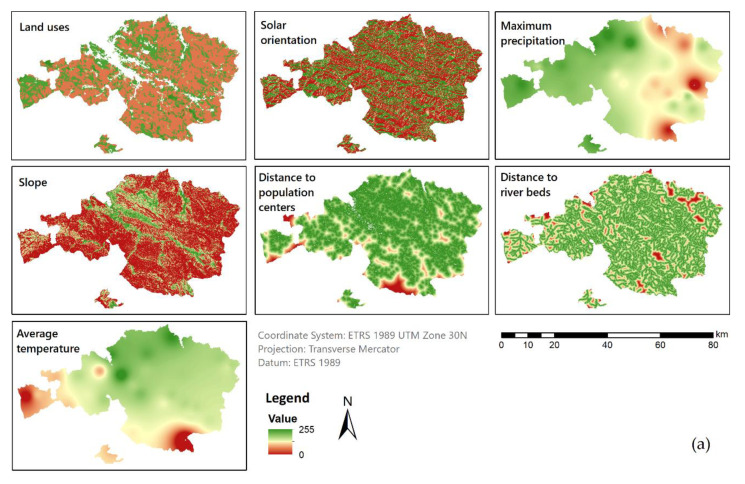

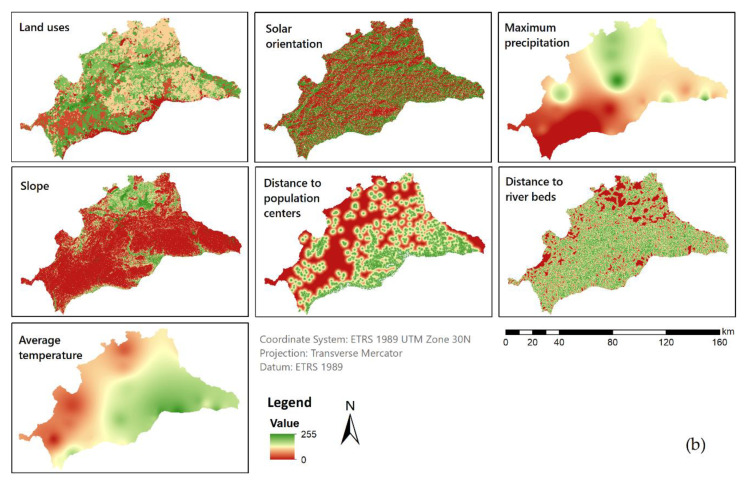
Map of the spatial distribution of standardized factors in Bizkaia, oceanic location (**a**) and Málaga, Mediterranean location (**b**). Normalized values range from 0 (not appropriate) to 255 (very appropriate) for each of the factors. Information sources listed in Table 2.

Two different regions located at Mediterranean and oceanic climates were analyzed using MCE. Previous to the application of the MCE method, a correlation analysis was performed between all the factors covered by the study. The results of correlation achieved a very low value (less than 0.3 in all cases); therefore, the factors were non-redundant. The maps corresponding to each of the factors were shown once normalized (0–255) for each study area with a raster grid definition of 25 × 25 m (Figure 4).

**Figure 5:**
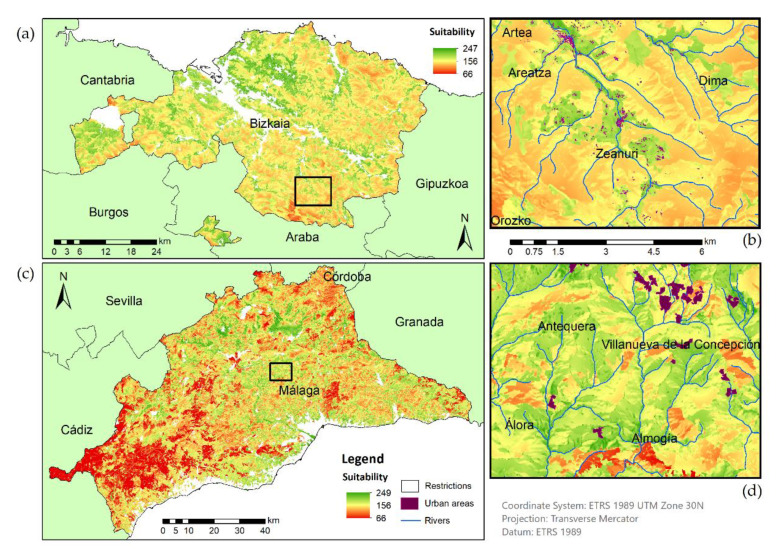
Mediterranean and oceanic locations suitability map for implementing METlands. On the left side a province scale map (**a**,**c**) and to the right a detailed view of the highlight squared areas, (**b**) Bizkaia and (**d**) Málaga. Information sources listed in Table 2.

Subsequently to the performance of the MCE for both locations, the suitability maps were obtained (Figure 5). Each pixel value indicates the portion of the territory suitable for the location of a METland. The higher values reveal the most suitable places for such treatment contrary to the lower values that point out less appropriate locations for implementing it. 

**Figure 6:**
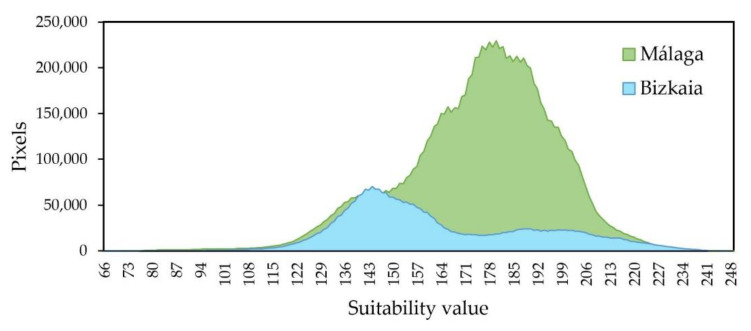
Distribution of pixels according to their suitability value. Málaga (Mediterranean location) and Bizkaia (oceanic location).

Regarding the suitability map at the provincial level (Figure 5), the main difference between the two provinces was the proportion of suitability areas. In the southwest of Málaga, a vast area of low suitability could be noticed, while in Bizkaia the low suitability areas were smaller. In the overall visual comparison between the two provinces it should be pointed out that Bizkaia had more intermediate values of suitability and Málaga had more abrupt changes. This perception could be due to the different area of each province; Málaga had a larger extension than Bizkaia. Another reason was regarding the frequency histogram of the suitability map because it had a very different distribution in each province. To verify this hypothesis, the distribution of parcels was represented according to their level of adequacy (Figure 6). 

Regarding the detail scale map, it should be noted that the oceanic location had a distribution of rural population widely dispersed in villages throughout the territory. While in Málaga, the population was grouped into larger settlements. Therefore, Málaga had an unequal population distribution between the coast and the inland area, characterized by a higher population density along the coast and medium/large urban settlements in other areas. As is noticed in Figure 5, the higher suitability values were congregated in areas close to dwellings and river channels. In addition, the maps showed that in both provinces the most suitable areas for METlands were located close to the population establishments or even isolated homes. This distribution proved the importance of treating the WW near the population centers. Moreover, in the mountain areas (in Málaga the southwest area and in Bizkaia the southern and the northeast area) lower suitability values were obtained, possibly due to the decrease of population cores and an increase of slope and unfavorable weather conditions.

In order to perform a more in-depth analysis of the values represented on the map, a histogram of suitability distribution was plotted (Figure 6). In Málaga, 6609 km^2^ (10,574,876 pixels) out of a total of 7307 km^2^ were suitable for METlands, representing 90.45% of the total. Whereas, the percentage of pixels suitable for such solutions in Bizkaia was 88.55% (3,090,217 pixels). The percentage of suitable area was higher compared with the study performed by Demesouka et al. (2013) [43] for nature-based WWT systems. However, it should be noted that Demesouka et al. (2013) [43] applied very prohibitive restrictions such as 5% of maximum slope, excluding from the analysis all the areas with higher slope and achieving lower rates of suitability. 

**Figure 7:**
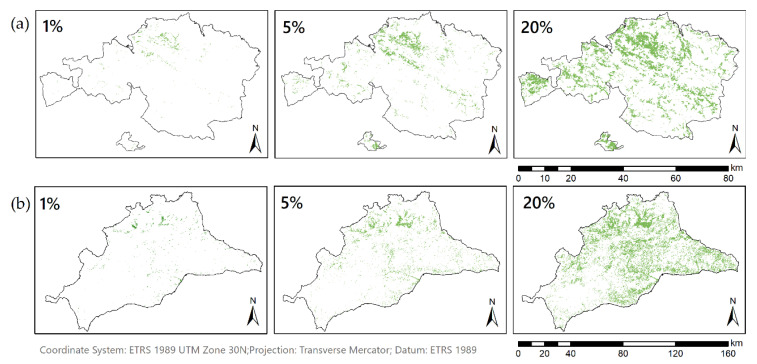
Boolean image of parcels with higher suitability in Bizkaia, oceanic location (**a**) and Málaga, Mediterranean location (**b**). Representing the 1%, 5% and 20% of the most suitable parcels of the entire province. Sources of information: IGN administrative divisions. Own elaboration.

On one hand, Málaga showed a normal distribution of the suitability, with the majority of pixels in the range of 160 to 200. In comparison, Bizkaia values were more regularly distributed where most of the pixels had adequacy between 135 and 160. On the other hand, Málaga achieved a percentage of suitable parcels slightly higher than Bizkaia, which might be due to the influence of the land uses map (Figure 4). Specifically, in the standardized map of land uses, Bizkaia presented a mean value of 89.17, while in Málaga it was 139.72, causing the great influence of land use in the final results of each province (Table 4). To test this hypothesis, the reclassified land use map had been overlaid on the suitability map. Thus, it could be verified that most of the areas of lower suitability mainly correspond to the land uses with a lower value in Table 1. Finally, in order to assess a detailed study of the data, 1%, 5% and 20% of pixels with higher suitability were analyzed (see Figure 7). 

The result of this application was some Boolean maps that represent a specific percentage of the best suitability areas out of the total. For Bizkaia, the 1% of pixels (30,902 pixels or 19.31 km^2^) with better suitability correspond to suitability values higher than 230, the 5% (154,510 pixels or 96.57 km^2^) at values greater than 216 and the 20% (618,043 pixels or 386.28 km^2^) to values greater than 192. For Málaga, the 1% of pixels (105,748 pixels or 66.09 km^2^) with higher suitability correspond to adequacy values greater than 219, the 5% (528,743 pixels or 330.46 km^2^) to values greater than 205 and the 20% (211,4975 pixels or 1321.86 km^2^) to values greater than 192.

To sum up, a map was obtained with the most suitable areas for the construction of METlands in both provinces (Figure 5). The results were better than expected and revealed optimal locations within the provinces analyzed. This analysis determined the most suitable areas on a large scale; however, further analysis could be performed for specific areas like specific municipality or areas with isolated houses. It should be noted that, for local analysis, it would be necessary to reduce the pixel size for a more accurate METland location. Regarding the comparison between the two provinces, similar results were obtained and the same methodology could be applied in other areas.

For replicability within other nature-based solutions, factors considered in the analysis should be adapted. The variables that determine the nature-based technology implementation must be listed (environmental, social and economic). Afterwards, experts should decide the importance of each variable in the final location for the system. The methodology proposed would eventually provide a suitability map from the area of study. We expect to help the stakeholder in the selection of new habitats to implement nature-based technologies for WWT, and set some guidance in the variables that specifically could influence operation of constructed wetlands. In this sense, some generic analysis had been conducted for WWT [41] and nature-based solutions [42], thus a specific analysis should be address for each technology and situation, taking into account the stakeholders interests. Additionally, some authors have previously analyzed different variables for specific technologies such as stabilization ponds [44] or restored wetlands [49,87]. 

### 3.2. Results of the GSA 

The results of the GSA for each of the model components (factors and weights) were compiled in Table 5. These results indicated that the variation in three of the factors contributed decisively in the model results, for all the study area. The determining factors were land uses, distance to population centers and distance to river channels. The meteorological criteria (average temperature and maximum precipitation) assumed a low contribution to the final result. These results obtained by the SA conclude that only a small number of the input factors were found to have a significant influence on the model results. This conclusion was corroborated in similar studies based on GIS-MCE models [31,32,52].

In Bizkaia, the order of importance of the criteria by the GSA was land use (38%), distance to the population centers (20%) and distance to river beds (20%). In Málaga, the order was land use (31%), distance to river beds (23%) and distance to population centers (22%). It could be highlighted that the proportion was not equal in both provinces since in Bizkaia land uses had more influence on the results as was anticipated in the discussion of the suitability maps. First-order sensitivity indices of the criteria of land use, distance to population centers and river beds were responsible for 78% of the output variability of the model. The influence of weights and other criteria was nearly negligible, confirming that the weights established for the variables were robust and the addition of small variations did not influence the final results of the model. Similar results were obtained by Vavatsikos et al. (2020) [45] with a 5% of variation for the weights and by Gómez-Delgado and Tarantola (2006) [52] with a 20% variation. Instead, for variations between 50% and 75% of the weights, some studies presented that the weight had a higher influence on the results than other criteria, without reaching the factors that represent the major source of variability [32,52]. 

In addition, the difference between the total effect sensitivity index (ST_i_) and the first-order sensitivity index (Si) is a measure of how much each factor is involved with the interaction with other factors in the model. For significant differences, the value should be greater than 0.2. In this analysis, the differences were never higher than 0.0121. Therefore, the variation in the results was due to the action of the factors individually and not in combination with the others. This circumstance was corroborated through the sum of all ST_i_, which were almost equal to 1 showing that potential interactions present in the model had no influence on the variability of outcome. 

From the results obtained it could be deduced that the most influential factors in the final model according to the SA were land uses, distance to the population centers and river beds. These results coincided with the initial factor classification; therefore, the weights were coherent and consistent. The methodology followed in the assessment was validated with these results, clarifying the relation between the input criteria and the final model. In both provinces similar MCE results were obtained, which could be interpreted as meaning that the established procedure was reproducible in other study areas, with similar purposes. Thus, to assess the robustness of the results a GSA should be performed to examine the effects that a change on the input might have on the model results. 

### 3.3. Optimization of Resources Based on the GSA

The GSA procedure implemented in the present study determined that the factors that most influenced the final model were the three mentioned above. Additionally, total indices identified unessential variables for model simplification, detecting those that were not important singularly or in combination with others. Thus, for the optimization of resources, a simplification in the number of factors was possible. Following the procedure implemented for other authors [52], which was summarized in Figure 3, the analysis was reproduced only with those three factors. The weights were redistributed and the same restrictions were considered. As a result, similar suitability maps were obtained for both locations, with a maximum variation in the suitability value of 29% (variation of 74 points in the scale of suitability over 247 of the maximum suitability value for Bizkaia). The range of variations produced between the first model and the optimized model was shown in Figure 6. 

**Figure 8:**
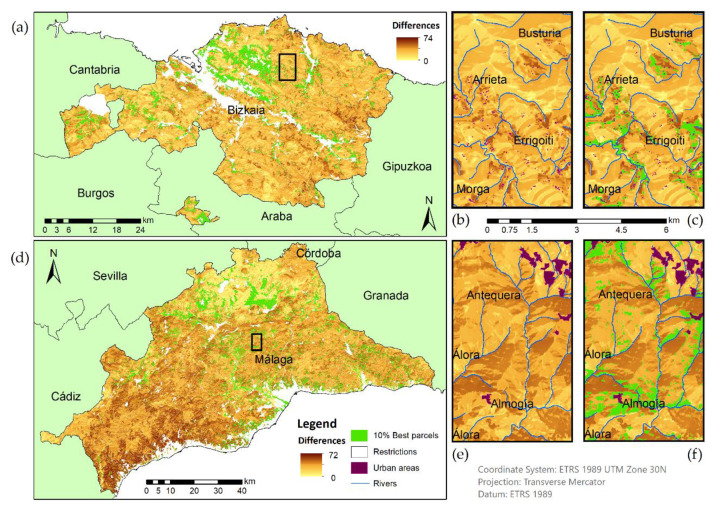
Suitability value differences between the first and second model in Bizkaia (oceanic location) and Málaga (Mediterranean location). On the left side a general map of both provinces is represented (**a**,**d**). On the right, a detailed view of the highlight squared areas (**b**,**e**) and with the overlap of the 10% of most suitable parcels (**c**,**f**). In Bizkaia, the maximum variation of suitability was 74 over 247 and in Málaga 72 over 249.

**Figure 9:**
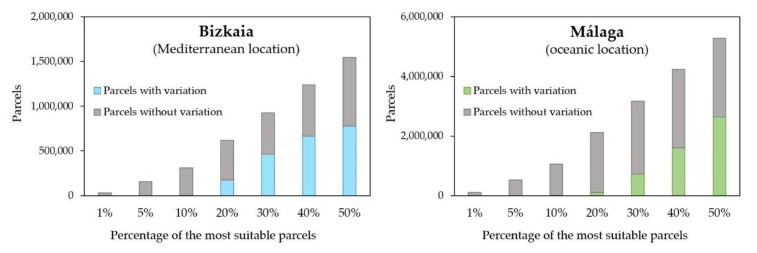
Relation between the most suitable areas of the MCE with all the factors and the areas with higher suitability variation among the models.

As could be noted in the maps, the main variations were associated with areas where the slopes and orientation (Figure 4) were responsible for most of the suitability value. Málaga presented a higher variation in the southwest as a result of the influence of temperature and precipitation factors in the first model and not in the second. At a local scale (Figure 8c,f) some patterns were discerned regarding the variation between models, for example, higher variations in the slopes that face south. Those parcels near river beds and populated areas were more affected regarding variations within models in Bizkaia (Figure 8b,c) in comparison with Málaga (Figure 8e,f); this is probably due to the different patterns of distribution of houses in the countryside (Bizkaia characterized by scattered single households and Málaga by villages). Once the main characteristics of the variations were analyzed, a specific study was implemented to relate both the most suitable areas from the first model and the higher fluctuation of the suitability values among models. The 10% of most suitable parcels in both provinces obtained the same suitability value with the first and the optimized model (Figure 9). Thus, the best locations for METland were not deeply influenced by the factors dismissed with the GSA. Indeed, we have produced a map that highlights the 10% of the most suitable parcels for METland (Figure 8). In the detail view, it could be noted that there is no overlap between these parcels and the areas with higher variability among the models (the original with seven factors versus the optimized with three factors). Thus, the most suitable parcels for METland are not affected by the reduction of factors in the second model. Once more, the disparity in the demographic distribution among provinces could be noted, Bizkaia presents a typical construction of single households disseminated through the area and Málaga shows small communities forming villages or small towns.

Regarding the similarities between the provinces, it could be acknowledged that the 10% of the most suitable parcels did not fluctuate in suitability value, but in the 20% of parcels such value did vary, with 28% in Bizkaia and 5% in Málaga. Our studies revealed that the maximum variation between the two models was 29% of the maximum suitability value. 

From the literature review, only one of the studies performed a simplification in the model by the results obtained with the GSA. Gómez-Delgado and Tarantola (2006) [52] executed the GIS-MCE model twice, first with the 11 factors and their respective weights and second with the three main criteria, observing that the best suitability distribution was not substantially modified. Those results were consistent with the conclusions obtained in the present study. Therefore, a simplification in the model could be achieved without affecting the results of the suitability map, at least in the most optimal location for METland. Finally, GSA could be addressed for identifying the most influential variables in other natural-based solutions. 

## 4. Conclusions

The combination of GIS and MCE methods is a powerful tool for solving planning problems in the field of wastewater treatment, providing enlightening information for decision-making in terms of resources or location of facilities. The selection of the input criteria for the MCE had implications in the rest of the study, where a poor choice or omission of information could produce significant changes in the analysis. The results clearly showed that a new variety of CW named METland was a versatile technology regarding the geographical location. In both locations, oceanic and Mediterranean, a large number of parcels (25 × 25 m) with suitability levels above 50% were found. Therefore, the implementation of METland had no major restrictions, with approximately 89% of suitable land for the construction. The GSA had provided information on how each of the factors influenced the final model and how just three of them responded to the 78% of the model variability (land use, distance to rivers and distance to population centers). Afterwards, the model was simplified with three such factors, and a similar suitability map was obtained in spite of managing less input resources.

From this study could be concluded that a proper characterization of the input factors and their frequency distributions is important in order to achieve reliable results. In addition, GSA techniques could be an effective tool for model simplification, in order to optimize the resources needed. By analyzing the total sensitivity indices, non-essential variables were identified, both independently and in conjunction with the rest, allowing for simplifications of the original model to be made. Furthermore, reproducing the methodology using the three most influential criteria achieved similar results (maximum of 29% of variation in the most unfavorable parcels) with a great optimization in the input data. It could be pointed out that the main variations were located in the areas where slope and temperature had greater influence. Additionally, the variations did not influence the 10% most suitable parcels for the location of METland. Thus, the procedure analyzed achieved satisfactory results, reducing the factors of the model to just the three most influential. Moreover, the current research is based on the precedents in the matter, seeking the greatest simplification of the problem, with the aim of making its replication simpler and minimizing resources (optimization of data acquisition and processing). 

Finally, it should be noted that although we have established a decision-making aid for implementing such constructed wetland following METland configuration, the same methodology could be applied to other CW configurations with different land footprints or to alternative nature-based technologies for treating wastewater. 

## Figures and Tables

**Table 1:** Reclassification of land uses according to their suitability for METland. (Source: CORINE).

Land Use *	Value
Forests	1
Permanently irrigated land	3
Rice fields	3
Permanent crops	4
Agro-forestry areas	4
Land principally occupied by agriculture, with significant areas of vegetation	6
Complex cultivation patterns	7
Annual crops associated with permanent crops	7
Non-irrigated arable land	8
Pastures	9
Scrub and/or herbaceous vegetation associations	9
Sparsely vegetated areas	10
Burnt areas	10

* The rest of the categories were included as restrictions.

**Table 2:** Description of factors, based on input data considered in the MCE.

Factor	Scale	Origin	Description	Reclassification	Normalization Function
Average temperature	1:25,000	AEMET, REDIAM and Provincial Council of Bizkaia	Average temperature interpolated based on the meteorological stations.	Growth range from 16 °C to 27 °C.	Linear monotonically increasing function (a = min., b = max.)
Maximum precipitation	1:25,000	AEMET, REDIAM and Provincial Council of Bizkaia	Maximum precipitation interpolated based on the meteorological stations.	The suitability decrease as higher maximum precipitation value.	Linear monotonically increasing function (a = min., b = max.)
Solar orientation	1:25,000	CNIG Download Center [73]	Land classification regarding the solar orientation based on the DEM.	The suitability increase in the south-oriented zones.	Symmetrical sigmoidal function (a = 45, b = 135, c = 225, d = 270)
Land use	1:100,000	CORINE Land Cover Project of IGN 2012	Reclassification of the land use database, according to high, medium o low level of suitability.	Land uses with special environmental or economic value are less suitable for the system.	Linear monotonically increasing function (a = 0, b = 10)
Distance to river beds	1:25,000	Water network database [74]	Distance to each river of the national water network in Spain.	Highest suitability values for places closer to the river systems.	Linear monotonically decreasing function (c = 25, d = max.)
Distance to population centers	1:25,000	INE, IGN, SIOSE [73]	Distance to inhabited areas considering from one household to cities. Avoiding non-residential buildings.	Areas closer to inhabited buildings are more suitable for construction.	Linear monotonically decreasing function (c = 25, d = max.)
Slope	1:25,000	CNIG Download Center [73]	Reclassification based on the percentage of slope suitable for the system.	Slopes between 0 and 15% have a linear suitability decrement.	Linear monotonically decreasing function (c = 0, d = 15)

AEMET: Spanish Agency of Meteorology; CNIG: National Center of Geographical Information; IGN: National Geographic Institute; INE: National Statistics Institute; MITECO: Ministry of Ecological Transition; REDIAM: Environmental Information Network of Andalucía.

**Table 3:** Assigning weights by hierarchy levels following the AHP method.

Criteria	Sub-Criteria	Weight
Environmental (w = 0.2)	Average temperature (w = 0.2)	0.04
	Maximum precipitation (w = 0.5)	0.1
	Solar orientation (w = 0.3)	0.06
Socio-economic (w = 0.8)	Land use (w = 0.25)	0.2
	Distance to river beds (w = 0.3)	0.24
	Distance to population centers (w = 0.3)	0.24
	Slopes (w = 0.15)	0.12

**Table 4:** Characteristics of the factors involved in the analysis.

		Oceanic Location		Mediterranean Location	
Criteria	Distribution	μ	σ	μ	σ
1. Land use	Discrete	89.17	96.89	139.72	81.89
2. Solar orientation	Discrete	106.85	113.17	120.92	114.73
3. Maximum precipitation	Beta	164.96	38.50	172.23	41.09
4. Slope	Discrete	48.99	73.50	48.97	79.31
5. Distance to population centers	Triangular	227.68	36.45	223.25	39.73
6. Distance to river beds	Triangular	224.01	43.37	226.97	45.32
7. Average temperature	Beta	174.15	31.71	125.72	48.68

**Table 5:** GSA results from the Sobol method.

	Oceanic Location		Mediterranean Location	
Factors	1° Order (S_i_)	Total (ST_i_)	1° Order (S_i_)	Total (ST_i_)
1. Land use	0.385049	0.394324	0.319312	0.321843
2. Orientations	0.055688	0.059115	0.071519	0.073032
3. Maximum precipitation	0.011405	0.011606	0.004406	0.004590
4. Slopes	0.089219	0.089736	0.101453	0.101764
5. Distance to population centers	0.201431	0.212660	0.222358	0.234555
6. Distance to riverbeds	0.200586	0.201577	0.233218	0.238349
7. Average temperature	0.002476	0.002083	0.003301	0.002738
w1	0.008062	0.017337	0.018631	0.021162
w2	−0.00126	0.002169	0.000649	0.002162
w3	0.005028	0.005229	0.007073	0.007256
w4	0.004267	0.004783	0.004141	0.004453
w5	0.029389	0.040617	0.034407	0.046604
w6	0.035728	0.036719	0.037700	0.042830
w7	−0.000625	−0.00102	0.0000458	−0.000517

The content in background color is displayed as the main factor.

## Data Availability

The data presented in this study are available on request from the corresponding author.

## Nomenclature

**Abbreviation**	**Definition**
AEMET	Spanish Agency of Meteorology
AHP	Analytical Hierarchy Process
CNIG	National Center of Geographical Information
CW	Constructed Wetland
DEM	Digital Elevation Model
GIS	Geographical Information Systems
GSA	Global Sensitivity Analysis
IGN	National Geographic Institute
INE	National Statistics Institute
MCE	Multi-Criteria Evaluation
MITECO	Ministry of Ecological Transition
MET	Microbial Electrochemical Technologies
OAT	One-At-a-Time approach
REDIAM	Environmental Information Network of Andalucía
SA	Sensibility Analysis
SIOSE	Spanish Land Cover Information System
WW	Wastewater
WWTs	Wastewater Treatments
